# Dendritic Spine and Synaptic Plasticity in Alzheimer’s Disease: A Focus on MicroRNA

**DOI:** 10.3389/fcell.2020.00255

**Published:** 2020-05-05

**Authors:** Edwin Estefan Reza-Zaldivar, Mercedes Azucena Hernández-Sápiens, Benito Minjarez, Ulises Gómez-Pinedo, Victor Javier Sánchez-González, Ana Laura Márquez-Aguirre, Alejandro Arturo Canales-Aguirre

**Affiliations:** ^1^Medical and Pharmaceutical Biotechnology Unit, CIATEJ, Guadalajara, Mexico; ^2^University Center of Biological and Agricultural Sciences, University of Guadalajara, Guadalajara, Mexico; ^3^Institute of Neurosciences, IdISSC, San Carlos Clinical Hospital, Madrid, Spain; ^4^University Center of Los Altos, University of Guadalajara, Guadalajara, Mexico; ^5^Preclinical Evaluation Unit, CIATEJ, Guadalajara, Mexico

**Keywords:** Alzheimer’s disease, dendritic spine, spine remodeling, synaptic plasticity, microRNA

## Abstract

Dendrites and dendritic spines are dynamic structures with pivotal roles in brain connectivity and have been recognized as the locus of long-term synaptic plasticity related to cognitive processes such as learning and memory. In neurodegenerative diseases, the spine dynamic morphology alteration, such as shape and spine density, affects functional characteristics leading to synaptic dysfunction and cognitive impairment. Recent evidence implicates dendritic spine dysfunction as a critical feature in the pathogenesis of dementia, particularly Alzheimer’s disease. The alteration of spine morphology and their loss is correlated with the cognitive decline in Alzheimer’s disease patients even in the absence of neuronal loss, however, the underlying mechanisms are poorly understood. Currently, the microRNAs have emerged as essential regulators of synaptic plasticity. The changes in neuronal microRNA expression that contribute to the modification of synaptic function through the modulation of dendritic spine morphology or by regulating the local protein translation to synaptic transmission are determinant for synapse formation and synaptic plasticity. Focusing on microRNA and its targets may provide insight into new therapeutic opportunities. In this review we summarize the experimental evidence of the role that the microRNA plays in dendritic spine remodeling and synaptic plasticity and its potential therapeutic approach in Alzheimer’s disease. Targeting synaptic deficits through the structural alteration of dendritic spines could form part of therapeutic strategies to improve synaptic plasticity and to ameliorate cognitive impairments in Alzheimer’s disease and other neurological diseases.

## Introduction

Nowadays, synaptic plasticity is considered a mechanism in which the structure and composition of synapses change according to determined neural activity (Frere and Slutsky, 2018; Oby et al., 2019). Collectively, it has been established that synaptic plasticity is determined by morphological and functional modifications of synapses as a response to activities that induce variations in the neuronal circuit, such as long-term potentiation (LTP) and long-term depression (LTD) (Ju and Zhou, 2018). At the cellular level, synaptic plasticity is mediated by structural changes (elongation, contraction, and shape changes), distribution, and function of the dendritic spines (Chidambaram et al., 2019).

Dendritic spines (DS) are small, highly dynamic protruding structures of the dendritic membrane where synapses occur (Chidambaram et al., 2019). These structures possess specialized subdomains with specific functions in synaptic transmission and plasticity, such as postsynaptic density (PSD), a complex composed by neurotransmitter receptors, mainly α-amino-3-hydroxy-5-methyl-4-isoxazole propionic acid (AMPAR) and N-methyl-D-aspartate receptors (NMDAR), scaffolding proteins, signal transduction molecules, ion channels, and cytoskeleton components that allow synaptic signal conversions into discrete biochemical responses that modify the spine morphology, receptor trafficking, and protein synthesis (Saneyoshi et al., 2010; González-Burgos, 2012). DS plasticity is crucial for the formation, maintenance and elimination of synapses. The assembly and remodeling of the neuronal circuit results from changes in density and properties of ionic channels and proteins that modulate the membrane conductance, as well as the cytoskeleton rearrangement (mainly actin filaments) that modifies the morphology and function of the DS (Fukazawa et al., 2003; Tada and Sheng, 2006; Fortin et al., 2012). As mentioned before, the electrophysiological mechanisms of LTP and LTD are effectors of DS plasticity due to the induction of large morphological and functional changes of DS. LTP is associated with a spine density increase, more stable spine heads and their components such as PSD (Segal, 2017). In contrast, LTD promotes DS contraction and density reduction (Hasegawa et al., 2015).

Currently, many neurological disorders are associated with aberrant changes in the neural connectivity and synaptic plasticity promoted by disruptions in DS shape, size, or density, suggesting that the DS dysfunction and the subsequent synaptic failure is a common and critical factor among these pathologies, particularly those involving deficits in information processing such as Alzheimer’s disease (AD) (Penzes et al., 2011; Dorostkar et al., 2015).

All structural and functional changes in DS involve gene regulatory networks controlling spine development, maturation and maintenance. In this context, microRNA (miRNA) have emerged as essential regulators of cellular processes to maintain an optimal synaptic plasticity during neuronal circuit formation and integration; a process that certainly implies the structural and functional modification of DS correlated with long-lasting changes in synaptic transmission (Hebert and De Strooper, 2009; Frere and Slutsky, 2018). This class of small non-coding RNA plays a significant role in the regulation of DS formation and stabilization during synaptic plasticity.

In summary, DS are considered a primary site of structural modification for synaptic plasticity. Alterations in these dynamic mechanisms are suggested to be directly involved in the development of neurological diseases like AD. Focusing on the miRNA, with its key roles in synaptic plasticity and controlling their expression, might be useful for cognitive capacity improvement. Thus, understanding the mechanisms involved in miRNA-mediated synaptic activity may provide insights into the development of new therapeutic strategies for neurological disorders such as AD.

## Synaptic Plasticity Impairment in Alzheimer’s Disease

AD is the most prevalent neurodegenerative disease among the elderly. This disease is characterized by synaptic plasticity dysfunction and a progressive decline in cognitive capacities. The AD hallmarks are the extracellular deposition and aggregation of amyloid-beta (Aβ) into amyloid plaques, and intracellular accumulation of neurofibrillary tangles of hyperphosphorylated tau protein. These protein depositions promote a synaptic dysfunction including synapse and neuronal loss (van de Weijer et al., 2020).

Several studies have correlated the DS loss and subsequent synaptic dysfunction with cognitive decline during disease progression (Scheff et al., 2007; Spires-Jones et al., 2007; Zhou et al., 2019). Although the molecular mechanisms underlying the neuronal and synaptic loss have not been fully described, some reports demonstrate that the excessive accumulation of Aβ is one of the main contributing factors (detailed reviewed Marttinen et al., 2018). Evidence suggests that Aβ-induced excitotoxicity results in the modification of electrophysiological processes in LTP and LTD, promoting a massive loss of DS due to a balance disruption of spine formation and elimination near the amyloid plaques (Hsieh et al., 2006; Shankar et al., 2007; Li et al., 2009). It has been shown that possibly through cytoskeleton alterations mediated by cofilin, p38/MAPK and calcineurin signaling, the Aβ oligomers could partially block the NMDAR signaling, reducing synaptically evoked Ca^2+^ influx, triggering events associated with LTD, such as increased AMPAR endocytic removal (Hsieh et al., 2006; Selkoe, 2008). In addition, it was reported that Aβ-toxicity promotes the overactivation of PAR-1/MARK and LKB-1 kinases, whose overactivity causes loss of PSD-95 function, leading to synaptic depression, DS, and synapses loss (Zhang et al., 2007).

Furthermore, the accumulation of Aβ triggers an impairment in the regulation of glutamate levels inducing cytotoxicity and the modification of DS morphology and NMDAR composition (Marttinen et al., 2018). The glutamate induces a dual effect, the elongation and contraction of the same group of spines. This depends of the calcium influx magnitude and duration (Korkotian and Segal, 1999). Prolonged exposure to a high glutamate concentration induces a rapid loss and reduction of DS size (Segal, 1995) and the NMDAR desensitization, reducing the Ca^2+^ influx and LTP induction (Shankar et al., 2007). In contrast, a low glutamate concentration increases the length of the DS (Segal and Andersen, 2000).

In contrast to the data about Aβ roles in synaptic loss, there are few reports about tau implication in synaptic dysfunction. Despite the fact that spine dynamics is mainly regulated by microfilament and actin-associated proteins, it has been demonstrated that microtubules also regulate DS morphology and synapses (Brandt and Bakota, 2017). In hippocampal rat neurons, Aβ exposition induces elevation of intracellular Ca^2+^ levels and aberrant activation of kinases which phosphorylate tau at the KXGS motif, leading to destabilization of dendritic microtubules; the impaired dendritic transport and Ca^2+^ homeostasis lead to the decay of DS and synapses (Zempel and Mandelkow, 2012). Additional studies show that hyperphosphorylated tau mediates NMDAR-dependent toxicity via Src-family tyrosine kinase Fyn, a kinase necessary for NMDAR phosphorylation and interaction with PSD-95 to a subsequent NMDAR stabilization (Ittner et al., 2010). Tau hyperphosphorylation leads to the Tau/Fyn/PSD-95/NMDAR complex dissociation disrupting LTP-required synaptic potentiation (Frandemiche et al., 2014), reducing the number of functional DS (Tracy and Gan, 2018). In addition, hyperphosphorylated tau missorting into somatodendritic compartments promotes NMDAR overactivation, followed by Ca^2+^ influx and cytotoxicity (Li et al., 2018). However, further functional studies are necessary to determinate the key cytoskeleton-associated components in the tau-induced toxicity.

These studies demonstrate the synaptic alterations mediated by Aβ and hyperphosphorylated tau protein toxicity. In this context, accumulation of Aβ and hyperphosphorylated tau could directly or indirectly promotes the weakening, inhibition of synapse strengthening and the further loss of synapses. Alterations in the DS cytoskeleton dynamics appear to accompany the synaptic alteration and memory in AD, in fact, such synaptic impairment seems to be a consequence of loss of DS and the impairment of LTD. Thus, understanding the molecular mechanism underlying AD synaptotoxicity may provide therapeutic targets for cognitive ability improvement.

## Roles of miRNA in Synaptic Plasticity

The miRNAs are a class of small non-coding RNAs of 18–22 nucleotides that act as posttranscriptional regulators of gene expression in virtually every cellular process. Their effects in gene silencing guide Argonaute proteins to target sites in the complementary 3′ untranslated region of mRNAs (Gebert and MacRae, 2019). In the brain, many miRNAs have been implicated in the neurogenic process such as the determination of neuronal fate, formation and stability of synapses, as well as synaptic plasticity modulation (Follert et al., 2014). Interestingly, miRNA expression increases under conditions which promote synaptic plasticity, for example LTD and LTP (Park and Tang, 2009).

Particularly in modulation of synaptic plasticity, miRNAs can regulate aspects such as dendritic outgrowth, spine morphology, and DS density (Table 1), contributing to the structural and functional organization of synapses as well as synaptic strength and excitability (Olde Loohuis et al., 2012; Ye et al., 2016).

**TABLE 1:** Summary of studies implying miRNA in synaptic plasticity.

miRNA	Target effects		Major findings	References
miRNA-206	BDNF	Downregulation	A dose-dependent decrease in the DS density.	Lee S. T. et al., 2012
miRNA-574	Nrn1	Downregulation	Cognitive impairment probably associated with reduction of neuritogenesis, DS stabilization, and maturation of synapses.	Li et al., 2015 Fujino et al., 2011
miRNA-34a	SIRT1 Syt-1 Stx-1A	Downregulation Downregulation Downregulation	Cognitive impairment associated with increasing levels of tau hyperphosphorylation, reduced dendritic trees and alteration of DS morphology and function.	Sarkar et al., 2019 Agostini et al., 2011
miRNA-30b	EphB2 SIRT1 GRIA2	Downregulation Downregulation Downregulation	Reduced basal synaptic transmission, impairment of LTP, impaired spatial learning and memory retention, as well as DS density reduction.	Song et al., 2019
miRNA-125b	Cdk5/p35 EphA4 GRIN2A	Upregulation Downregulation Downregulation	Upregulation of MAPK signaling, resulting in tau hyperphosphorylation. Formation of long and narrow DS filopodia-like, with a subsequently synaptic transmission weakening.	Banzhaf-Strathmann et al., 2014 Edbauer et al., 2010
miRNA-134	Limk1 CREB	Downregulation Downregulation	Marked DS density and DS size reduction, impairment of LTP, resulting in a weak synaptic transmission.	Schratt et al., 2006 Rajasethupathy et al., 2009
miRNA-29a/b	Arpc3	Downregulation	Reduced probability of actin branch formation, reducing the mushroom-shaped DS formation, and DS head enlargement, a fundamental step in synaptic maturation.	Lippi et al., 2011
miRNA-135	CPLX1 CPLX2	Downregulation Downregulation	Impairment of the postsynaptic exocytosis of AMPA receptors leading to DS retraction long-lasting DS shrinkage.	Hu et al., 2014
miRNA-124	CREB	Downregulation	Constrains serotonin-induced long-term synaptic plasticity via CREB. miRNA-124 inhibition promotes the short-term conversion to long-term synaptic facilitation, enhancement of serotonin-induced synaptic plasticity.	Rajasethupathy et al., 2009
miRNA-485	GRIA2 PSD-95	Downregulation Downregulation	Impairments of synaptic function and synapse number decreasing. Reduction of mature DS density and increased appearance of long and thin immature spines.	Cohen et al., 2011
miRNA-138	LYPLA1	Downregulation	Triggering of a high RhoA signaling, inducing DS shrinkage and reduction of synaptic activity.	Siegel et al., 2009
miRNA-132	ARHGAP32 MMP-9 CREB BDNF	Downregulation Downregulation Upregulation Upregulation	Induction of activity-depend DS formation, formation of mushroom spines, increases DS head widening, and their subsequent maturation, a process involved in the potentiation of synaptic plasticity.	Wayman et al., 2008 Jasinska et al., 2016 Mellios et al., 2011
miRNA-188	NRP2	Downregulation	Increase the DS development, synaptic structure, and mEPSC frequency. Repression of DS density loss.	Lee K. et al., 2012.
miRNA-191	TMOD2	Downregulation	Decreased contraction of DS and their subsequent elimination in LTD.	Hu et al., 2014
miRNA-218	GRIA2	Upregulation	Increasing of the amplitude of synaptic currents and the formation of thin DS.	Rocchi et al., 2019 Torres-Berrio et al., 2019

*Abbreviations in the table were illustrated in the text.

## miRNA Associated to Ad-Synaptic Plasticity Impairment

Different studies on AD revealed that altered miRNAs levels including the miRNA-101, miRNA-106, miRNA124, miRNA-9, miRNA-134, miRNA-132, miRNA-29a/b, miRNA-107, miRNA-206, and miRNA-146 (Candido et al., 2019), play a critical role in the phenotypic changes during disease progression, particularly the production of Aβ, hyperphosphorylated tau, BACE1, and gamma-secretase expression and ADAM-secretase inhibition (detailed reviewed in Reddy et al., 2017). Here, we reviewed those particularly involved in synaptic and DS plasticity. For instance, the miRNA-206 overexpression in Tg2576 mice that negatively regulates BDNF (UniProt code P23560), a neurotrophin with the ability to modulate cognitive function, enhances neurogenesis, and facilitates LTP. This overexpression contributes to AD pathology through BDNF downregulation. In contrast, miRNA-206 inhibition enhances synaptophysin expression and increases the hippocampal neurogenesis (Lee S. T. et al., 2012). In APP/PS1 mice, the hippocampal synaptic loss is accompanied by miRNA-574 overexpression, whose effects are mediated probably by Nrn1 (UniProt code Q9NPD7) inhibition (Li et al., 2015). Nrn1 is a neurotrophic factor that promotes neuritogenesis, spine stabilization, and formation and maturation of synapses (Fujino et al., 2011). Besides the synaptic loss, miRNA-574 overexpression is correlated with the cognitive impairment in this model.

In a miRNA-34a overexpression transgenic mice, the appearance of cognitive decline and AD neuropathology are sooner than in a single Tg-AD mice, 3xTg-AD mice, and even 5xTg-AD mice (Sarkar et al., 2019). This study showed that SIRT1 (UniProt code Q96EB6) is the main target of miRNA-34a. SIRT1 is necessary for cognitive function and synaptic plasticity-dependent of CREB (UniProt code P16220) and BDNF (Gao et al., 2010). SIRT1 inhibition by miRNA-34a increases tau phosphorylation levels (Sarkar et al., 2019). Additionally, miRNA-34a overexpression alters the spine morphology and function by targeting the synaptic proteins Syt-1 (UniProt code P21579) and Stx-1A (UniProt code Q16623) (Agostini et al., 2011). In a 5xFAD APP transgenic mice and AD patients’ brains, it was evidenced that an elevated expression of miRNA-30b significantly downregulates ephB2 (UniProt code P29323), SIRT1, and GRIA2 (Uniprot code P42262) – molecules necessary for maintaining synaptic integrity. Overexpression of miRNA-30b in the hippocampus impairs basal synaptic transmission, LTP, learning, and memory, as well as reduction of DS density (Song et al., 2019).

In an *in vitro* neuron culture, miRNA-125b overexpression leads to elevated Cdk5/p35 (UniProt code Q15078) expression and MAPK signaling, which results in Tau hyperphosphorylation at multiple sites. Also, miRNA-125b injection into the hippocampus impairs learning in mice (Banzhaf-Strathmann et al., 2014).

Despite the fact that the precise pathological mechanisms underlying synaptic impairment remain unclear, the evidence suggests that dysregulation of miRNA in AD is closely associated with the negative regulation of synaptic capacity and cognitive decline. The dysregulation of miRNA in AD and its implications to cognitive deficit requires further studies.

## Regulation Dendritic Spines Plasticity by miRNA

**FIGURE 1:**
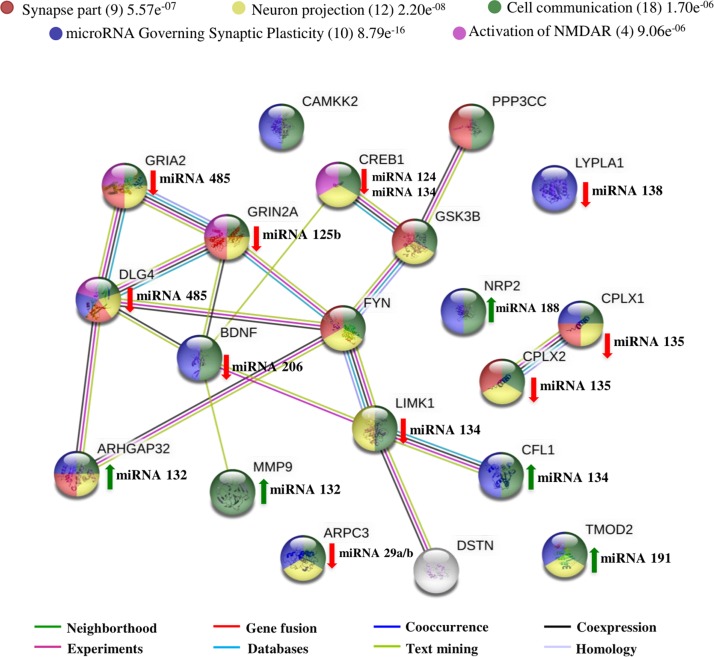
Interactome of polypeptides regulated by miRNAs found concerning synaptic plasticity and spine dynamic morphology. UniProtKB accession numbers were submitted to the String program to identify the predicted functional network. Colored stripes represent different evidence for each identified interaction. The polypeptides which are Synapse part (19 members), involved in Neuron projection (22 members), Regulation of cytoskeleton organization (16 members), involved in microRNA-mediated synaptic plasticity (14 members), regulation of neuron projection development (13 members) and protein binding (33 members) were denoted by yellow, pink, red, dark green, blue and green nodes respectively. miRNAs with green up arrows have a positive effect in synaptic plasticity while miRNA with red down arrows have a negative effect. All proteins show statistical significance (*p*-Value ≤ 0.05) in the different biological processes that were related.

Currently, there are few miRNAs described with a specific function in the regulation of spinogenesis and DS morphology (Figure 1). There are miRNAs with negative effects in the DS plasticity, for example, the miRNA-134, whose effect is mediated by Limk1 (UniProt code P53667) inhibition, a kinase that promotes spine actin polymerization by inhibiting ADF/cofilin. Therefore, the overexpression of miRNA-134 causes a marked DS density reduction resulting in a weak synaptic transmission (Schratt et al., 2006). Another miRNA with a negative cytoskeleton regulation is the miRNA-29a/b cluster; miRNA-29a/b expression regulates the DS morphology through Arpc3 (UniProt code O15145), a subunit of the actin ARP2/3 nucleation complex, reducing the probability of actin branch formation, a fundamental step in DS maturation (Lippi et al., 2011). miRNA-125b negatively regulates synaptic plasticity by inhibition of GRIN2A (UniProt code Q12879) and possibly EphA4 (UniProt code P54764); miRNA-125b overexpression induces formation of long and narrow DS filopodia-like, with a subsequently synaptic transmission weakening. There is a positive correlation between the miRNA-125b expression and the cognition decline (Edbauer et al., 2010).

Moreover, it has been reported that miRNAs control the DS and synapse stabilization. In an LTD study, it was reported that miRNA-135 negatively regulates CPLX1 (UniProt code O14810) and CPLX2 (UniProt code Q6PUV4), complexins implicated in the late step of vesicles exocytosis, which inhibition reduces the postsynaptic exocytosis of AMPA receptors leading to DS retraction (Hu et al., 2014). The miRNA-124 and miRNA-134 have been associated with synaptic plasticity modification by CREB regulation. The reduction of miRNA-124 expression enhances synaptic plasticity allowing the increase of CREB-dependent transcription (Rajasethupathy et al., 2009). In the same way, miRNA-134 inhibition increases the production of BDNF by enhancing CREB activity (Gao et al., 2010). In addition, overexpression of miRNA-485 reduces DS density by GRIA2 inhibition (UniProt code P42262) along PSD-95 (DLG4, UniProt code P78352) clustering; however, the mechanism by which this miRNA produces the density reduction is unknown (Cohen et al., 2011). In another study, it was reported that miRNA-138 regulates DS growth by LYPLA1 inhibition (UniProt code O75608), an enzyme with a function in protein depalmitoylation. The increase in palmitoylation of the cysteine residues and their subsequent membrane location triggers a high RhoA signaling, which induces DS shrinkage with a reducing synaptic activity (Siegel et al., 2009).

On the other hand, there are miRNAs with a positive regulation on DS plasticity. For instance, miRNA-132, the best-studied miRNA in the synaptic plasticity context induces the formation of spines inhibiting ARHGAP32 (UniProt code A7KAX9), a member of the Rho GTPase family (Wayman et al., 2008). Subsequently, it was reported that miRNA-132 inhibits the extrasynaptic MMP-9 (UniProt code P14780), whose overexpression promotes the formation of long, thin and immature DS; MMP-9 inhibition by miRNA-132 promotes DS head widening, a process involved in the potentiation of synaptic plasticity (Jasinska et al., 2016). Unlike as miRNA-124 and miRNA-134, miRNA-132 is required for CREB-mediated dendritic growth and spine formation, particularly the formation of mushroom spines and their subsequent maturation (Mellios et al., 2011). In a recent study, the upregulation of miRNA-132 by an enriched environment enhanced hippocampal synaptic plasticity, and prevented the LTP impairments induced by Aβ oligomers (Wei et al., 2020). Interestingly, the miRNA-132 overexpression in transgenic models, promotes an excessive DS growth (Hansen et al., 2013), limiting the proper functioning of neurons and inducing alterations of LTP and LTD (Scott et al., 2012).

In another study, it was described that miRNA-188 overexpression selectively inhibits NRP2 (UniProt code O60462), a semaphorin 3F receptor that induces neuronal growth cones repression, and is capable of disturbing the DS and synaptic structure leading to a significant decrease of DS density. Therefore, when miRNA-188 inhibits NRP2, the reduction in DS density is reverted (Lee K. et al., 2012). In LTD, miRNA-191 decreased the elimination and contraction of DS by inhibiting TMOD2 (UniProt code Q9NZR1), a protein that promotes the depolymerization of actin (Hu et al., 2014). The miRNA-218 enhances the expression of GRIA2 subunit of AMPAR, increasing the amplitude of synaptic currents (Rocchi et al., 2019) and the formation of thin DS (Torres-Berrio et al., 2019).

Together, all this evidence shows the miRNAs regulatory roles in DS morphogenesis, both in pathological and physiological conditions. Although further research is necessary to clarify the crosstalk between these molecules and their targets, understanding all the miRNA-related effects could be used to develop protective strategies against the functional and behavioral alterations induced by chronic stress conditions such as AD.

## Protein-Protein Interaction Networks and Functional Enrichment Analysis

We performed a literature search in databases including Web of Science, PubMed and Google Scholar. The keywords chosen included “microRNA,” “Alzheimer’s Disease” with either “dendritic spine,” “synapse,” and “synaptic plasticity.” With the information extracted from the selected articles we generated a figure composed of a network of known and predicted protein-protein interactions using the STRING program (Szklarczyk et al., 2017) and reported miRNAs targets. The interactome network represented in Figure 1, describes a minimum required interaction score of 0.70 (high confidence) between proteins, highlighting in red the biological processes of regulation of cytoskeleton organization with the interaction between 16 members with a false discovery rate (FDR) of 1.89e^–14^. In blue is shown the regulation of neuron projection development with 13 members and an FDR of 4.59e^–11^. In green, is shown the protein binding as the main molecular function with 33 members and an FDR of 4.23e^–10^. In pink is shown all the 22 members which are part of the neuron projection and in yellow the 19 members localized/involved in the synapse with an FDR of 5.91e^–16^ and 6.39e^–15^, respectively. Finally, in dark green, are shown the 14 members which are regulated by miRNA implicated in synaptic plasticity with an FDR of 1.14e^–20^.

Here, we show that most of the reported miRNAs affect the protein binding property (green nodes) of their targets. This promotes incapacity in assembly complexes required for cytoskeleton modification (red nodes) as reported by Lippi et al., 2011, or in the case of GRIN2A, the interaction with DLG4 to NMDAR stabilization and LTP induction, a process associated with the formation and maturation of mushroom DS (Mellios et al., 2011). In the synapse (yellow node), miRNAs such as miRNA-30b, miRNA-125b, and miRNA-485 interfere with the activation of NMDAR and AMPAR by inhibiting glutamate receptor subunits or impairment of AMPAR, and NMDR exocytosis. Impairments in AMPAR and NMDAR stabilization and activation results in unfavorable changes in DS morphology that blocks LTP activation. Interestingly, most of the miRNAs induce changes in the synaptic capacity by destabilization of the DS cytoskeleton, and although it was not discussed here, the inhibition of different proteins by particular miRNA could simultaneously impact other components involved in plasticity (Neighborhood and experiments stripes). In contrast, overexpression of miRNA-132, miRNA-188, and miRNA-191 could enhance DS formation and maturation by inhibiting enzymes with actin depolymerization functions and neuronal growth cone repressions.

This predicted protein-protein interactions network emphasizes the critical role of miRNA in the synaptic plasticity of normal neural function. This kind of bioinformatic analysis allows for the identification of potential pathways linking the pathological synaptic activity to miRNA function and in this way, the design of therapeutics strategies.

Nowadays, with sophisticated tools for miRNA expression screening and target prediction we can identify key miRNAs in certain processes (e.g., DS cytoskeleton destabilization). However, these computational predictions have a low accuracy rate and could include false-positive non-functional targets and miss some true-positive targets. Therefore, it is essential to validate these predictions with *in vivo* and *in vitro* experiments, and thus, determine their suitability for therapeutic strategies. Despite complications around miRNA therapeutics, such as a poor understanding of the stoichiometric relationship between a specific miRNA and the copy of its targets, the ubiquitous miRNA nature, miRNA site-specificity, and off-target effects (Bonneau et al., 2019; Bajan and Hutvagner, 2020), miRNA technologies have been applied successfully as an experimental therapy in basic research, as demonstrated by all the studies reviewed.

## Conclusion and Perspectives

Synaptic dysfunction is a common and critical factor among many neurological disorders. Synaptic failure as a consequence of DS loss has been associated with the cognitive impairment in pathologies such as AD. In this context, it has already demonstrated that miRNAs play essential roles in the synaptic regulation and neural survival, thus influencing cognitive function in both physiological and pathological conditions.

Even though the disease progression interference through miRNA regulation seems to be a promising therapeutic strategy, it is necessary a deep insight into the miRNA signaling to understand the mechanisms that rule processing, stability, and activity of these molecules in both physiological and pathological conditions. Furthermore, the identification of signaling pathways that strengthen synaptic transmission and synaptogenesis is required to design strategies for rational synaptic plasticity structural and functional modification. Also, it is crucial to determine the effects of synaptic modification in existing neural circuits before the development of miRNA treatment strategies.

Combining high-throughput identification and functional assays to identify miRNA activity with the application of different technologies such as patient-specific IPSC-derived neuron cultures for neurodegenerative disease modeling, plus next-generation sequencing, represents an excellent experimental system to evaluate the relative expression, function, and target transcripts of particular miRNAs at the cellular level. Moreover, with the development of advanced techniques like two-photon laser scanning microscopy combined with glutamate caging, and super-resolution spine imaging and humanized cell models, we could determine the precise neuroanatomical changes of DS in several conditions and understand the synaptic connectivity and plasticity processes, allowing for the development of new therapeutic strategies for AD and other neurological disorder treatments.

## Data Availability Statement

All datasets generated and analyzed for this study are included in the article/supplementary material.

## Author Contributions

ER-Z, MH-S, BM, UG-P, VS-G, AM-A, and AC-A equally contributed to the literature research, writing, and correcting of this perspective.

## Conflict of Interest

The authors declare that the research was conducted in the absence of any commercial or financial relationships that could be construed as a potential conflict of interest.
